# Impact of COVID-19 pandemic and the lockdown on invasive meningococcal disease

**DOI:** 10.1186/s13104-020-05241-9

**Published:** 2020-08-27

**Authors:** Muhamed-Kheir Taha, Ala-Eddine Deghmane

**Affiliations:** Invasive Bacterial Infections Unit and the National Reference Centre for Meningococci and Haemophilus influenzae, Paris, France

**Keywords:** Infection, Invasive meningococcal disease, Respiratory presentations, SARS-CoV-2, COVID-19, Typing

## Abstract

**Objective:**

Few data are available on the association between SARS-CoV-2 and secondary bacterial infections. Such an association was described for flu and invasive meningococcal disease (IMD). We aimed exploring such a correlation between COVID-19 and IMD as well as the impact of the lockdown on IMD.

**Results:**

We compared IMD cases received at the French National Reference Centre for meningococci and *Haemophilus influenzae* that are sent as part of the mandatory reporting of IMD. We compared these data during the period 01 January-15 May 2020 to those from the same period in 2018 and 2019. IMD cases that were associated with respiratory presentations significantly increased in 2020 compared to 2018 (*P *= 0.029) and 2019 (*P *= 0.002), involved elderly and were due to unusual isolates. However, IMD cases due to hyperinvasive isolates decreased during the lockdown. Enhancing IMD surveillance and anti-meningococcal vaccination in elderly should be addressed.

## Introduction

*Neisseria meningitidis* (Nm) is a Gram negative bacterium with airborne inter-human transmission. Nm is carried asymptomatically in the nasopharynx with 10% carriers in the general population [1]. However, Nm is also responsible for invasive meningococcal disease (IMD) that is dominated by septicaemia and meningitis [2]. Nm is highly diverse due to frequent DNA transfer between isolates followed by recombination and allelic exchanges [3]. Genetic typing is crucial for epidemiological surveillance and is performed by DNA sequencing using multilocus sequence typing (MLST) and whole genome sequencing (WGS). The isolates are classified into genetic lineages called clonal complexes (CC) and those that are most frequently associated with IMD are referred to as hyperinvasive CC but other diverse CC are more associated with carriage [1]. Risk factors to develop IMD include bacterial virulence factors, host factors such as complement deficiencies and environmental factors such as viral infections where IMD can be associated with respiratory manifestations such as bacteremic pneumonia [2, 4]. The association of viral infections and secondary bacterial infections has been described such as the association between flu and secondary bacterial infections including IMD [5, 6]. During the 1918 pandemic flu, fatality records suggested large impact of secondary bacterial infection [5]. Measures impacting airborne transmitted agents such as social and physical distancing are expected to reduce both flu and Nm transmissions and therefore to reduce the incidence of IMD. Indeed, it has been observed one century ago that bed distancing of 3 feet in military barracks reduced the risk of IMD outbreak among new recruits [7].

The implementation of lockdown to control COVID-19 pandemic may therefore interfere with the epidemiology of IMD. Moreover, sepsis was also observed as a common complication during COVID-19 [8]. We therefore checked the impact of the lockdown period during the COVID-19 pandemic in France on IMD cases.

## Main text

**Fig. 1:**
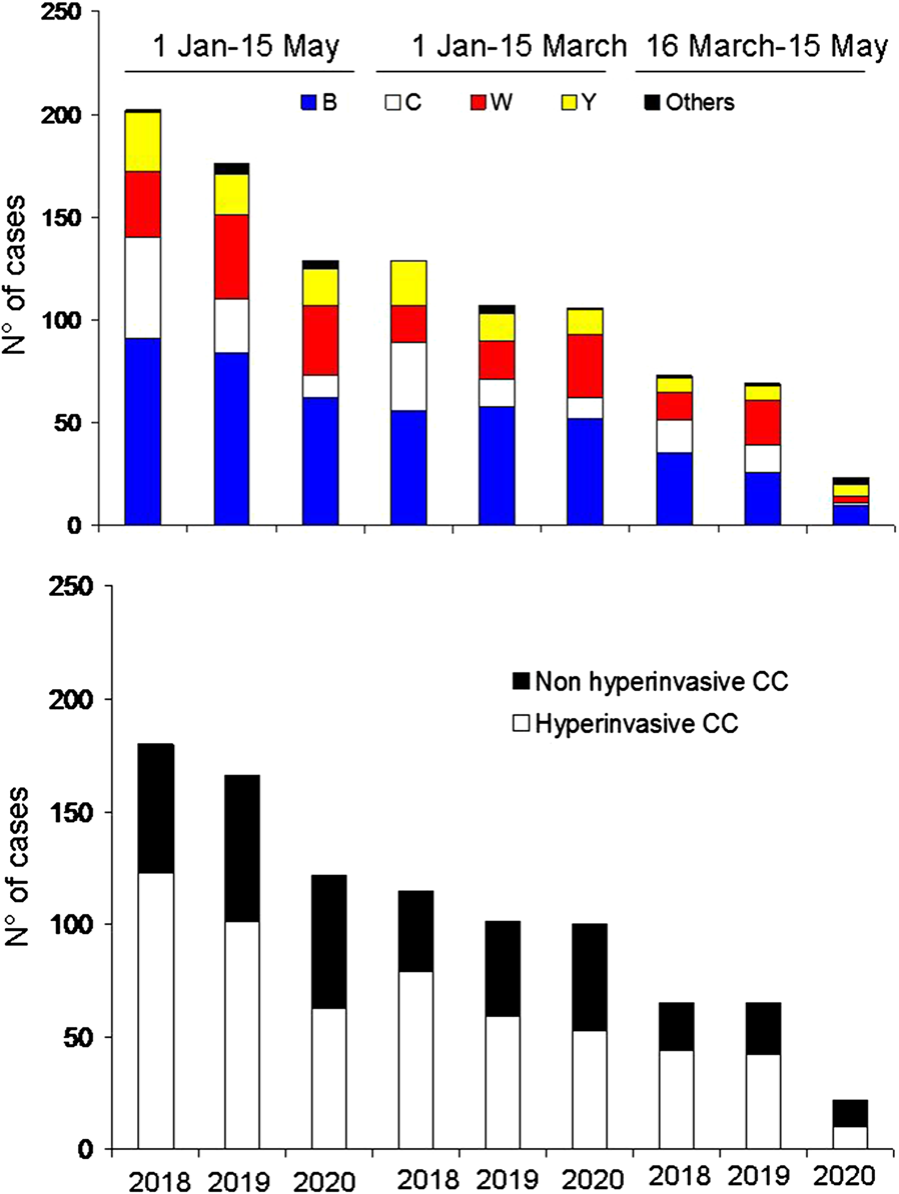
Distribution of isolates responsible for IMD according to serogroups (above) and genetic lineages (down) during the three periods of 2018–2020 as indicated. Hyperinvasive CC are CC11, CC32, CC41/44 and CC269 [3]

We screened the database of the French national reference centre for meningococci and *Haemophilus influenzae* (NRCMHi) for biologically confirmed IMD cases (meningococci detected by culture and/or PCR from a normally sterile site) and compared data for the period between 01 January and 15 May of 2018, 2019 and 2020 using the Chi squared test. Meningococcal isolates are sent to the NRCMHi for full typing (MLST and WGS) as part of the mandatory reporting of IMD [9]. A total of 507 cases of IMD were received at the NRCMHi for the 3 years (202 in 2018, 176 in 2019 and 129 in 2020). The global numbers of cases were decreasing since 2018. The first raison was most likely the introduction of the mandatory vaccination against group C (NmC) meningococci in January 2018. IMD due to serogroup C decreased from 49 cases in 2018 to 11 cases in 2020 (*P *= 0.002 Chi Square Test) while the changes in the other serogroups was not significant. The number of IMD cases did not significantly differ between 2018, 2019 and 2020 before the period 16 March–15 May (Table 1 and Fig. 1). However, when the analysis focused on the lockdown period (16 March 2020–15 May 2020), the decrease of all IMD cases (regardless the serogroup) from 2018 was significant with only 23 IMD cases during the lockdown period in 2020 versus 2018 and 2019 with 73 IMD and 68 (*P *= 0.002 and 0.001 Chi Square Test) respectively (Table 1 and Fig. 1). Moreover, the decrease during the lockdown, seemed to mainly involve IMD cases due to serogroups B and C and W but not IMD due to serogroup Y and other unusual serogroups or non-serogroupeable isolates which did not decrease significantly and which proportions increased during the lockdown 2020 (Table 1 and Fig. 1). A special attention was drawn to IMD cases due to serogroup W as they were continuing to increase in 2020 before the lockdown. The emergence of highly transmissible and hyperinvasive serogroup W isolates of the clonal complex CC11 was described in France as in other countries in Europe since 2013 [10, 11]. The serogroup W isolates showed a sharp decrease during the lockdown (3 cases in 2020 during the lockdown versus 14 and 21 cases during the corresponding period in 2018 and 2019; *P *= 0.005 and *P *= 0.001 respectively using Chi Square Test). Moreover, The decrease contrasted with the increasing number of IMD cases due to serogroup W before the lockdown period in 2020 compared to the same period in 2018 and 2019 (31 cases before the lockdown in 2020 versus 18 and 20 cases for the corresponding period in 2018 and 2019, *P *= 0.1 and *P *= 0.027 respectively using Chi Square Test). The decrease during the lockdown involved mainly the highly transmissible and hyperinvasive isolates belonging to the clonal complex CC11 [11] that almost disappeared.

**Table 1:** Characteristics of IMD cases

		2018			2019			2020		
		01 Jan–15 March	16 March–15 May	Total	01 Jan–15 March	16 March–15 May	Total	01 Jan–15 March	16 March–15 May	Total
IMD cases without respiratory presentations		118	71 *P *= 0.001*	189	103	66 *P *= 0.001	169	93	18	111
Sex ratio (M/F)		1 (59/59)	1.09 (37/34)	1.03 (96/93)	0.72 (43/60)	0.83 (30/36)	0.78 (73/96)	0.79 (41/52)	1 (9/9)	0.82 (50/61)
Median Age (Interquartile range Q1–Q3) y		21.3 (2.4–46.6)	20.6 (2.5–42.8)	20.7 (2.4–43.2)	25.1 (2.9–56.7)	18.7 (1.4–52.8)	21.1 (2.4–54.3)	21.4 (5.3–56.9)	15.9 (1.7–47.9)	20.5 (3.2–55.8)
Serogroup	B	56	35	91	58	26	84	49	10	59
	C	30	15	45	13	13	26	10	0	10
	W	17	13	30	19	20	39	24	3	27
	Y	15	7	22	10	6	16	9	4	13
	Others	0	1	1	3	1	4	1	1	2
Clonal complexes	Hyperinvasive clonal complexes	75	42	117	59	42	101	49	9	58
	Non-hyperinvasive clonal complexes	29	21	50	39	21	60	39	9	48
IMD cases with respiratory presentations		11	2	13, *P *= 0.029	5	2	7, *P *= 0.002	13	5	18
Sex ratio (M/F)		0.22 (2/9)	0 (0/2)	0.18 (2/11)	0.25 (1/4)	0 (0/2)	0.17 (1/6)	0.86 (6/7)	0.25 (1/4)	0.64 (7/11)
Median Age (Interquartile range Q1–Q3) y		67.5 (60.1–82.5)	89.2 (85.3–93.1)	74 (62.9–84.3)	82.2 (50.4–84.8)	47.0 (0.7–93.4)	82.2 (33.5–85.7)	86.8 (66–93.0)	50.1 (15.7–83.1)	70.7 (52.9–93.0)
Serogroup	B	0	0	0, *P* = 0.036	0	0	0	3	0	3
	C	3	1	4, *P* = 0.003	0	0	0	0	1	1
	W	1	1	2	1	1	2	7	0	7
	Y	7	0	7	3	1	4	3	2	5
	Others	0	0	0	1	0	1	0	2	2
Clonal complexes	Hyperinvasive clonal complexes	2	2	4	0	0	0	4	1	5
	Non-hyperinvasive clonal complexes	7	0	7	3	2	5	8	3	11
All IMD		129	73 (*P *= 0.002)	202	108	68 (*P *= 0.001)	176	106	23	129
Sex ratio (M/F)		0.89 (61/68)	1.03(37/36)	0.94 (98/104)	0.69 (44/64)	0.79 (30/38)	0.73 (74/102)	0.80 (47/59)	0.77 (10/13)	0.80 (57/72)
Median Age (Interquartile range Q1–Q3) y		22.2 (2.8–58.3)	20.8 (4.5–44.2)	21.5 (2.8–51.7)	30.4 (4.9–59.6)	18.7 (1.4–53.1)	22.3 (2.5–56.6)	27.7 (6.8–65.9)	19.8 (3.0–50.4)	25 (5.3–64.1)
Serogroup	B	56	35	91	58	26	84	52	10	62
	C	33	16	49	13	13	26	10	1	11
	W	18	14 (*P *= 0.005)	32	20	21 (*P *= 0.001)	41	31	3	34
	Y	22	7	29	13	7	20	12	6	18
	Others	0	1	1	4	1	5	1	3	4
Clonal complexes	Hyperinvasive clonal complexes	79	44	123	59	42	101	53	10	63
	Non-hyperinvasive clonal complexes	36	21	57	42	23	65	47	12	59

*Chi squared test. The test compared the data for the years 2018 or 2019 to the corresponding data in the year 2020. Only significant *P* values are shown

The MLST typing data were obtained for 468 cases (92% of all IMD cases of this report; 180 for 2018, 166 for 2019 and 122 for 2020). The distribution of genetic lineages differed mainly during the lockdown period in 2020 compared to the same period in 2018 and 2019 with lower proportion (although not significantly), of hyperinvasive genetic lineages in 2020 during the lockdown period (45% in 2020 versus 68% and 65% for the same period in 2018 and 2019 respectively). This proportion did not differ outside the period corresponding to the lockdown for the 3 years (Table 1 and Fig. 1). The genotypes that did not changed in 2020 were CC23 and the isolates belonging to the unassigned clonal complexes (UA). These isolates were frequently of serogroup Y (Additional file 1: Table S1). Indeed, IMD due to serogroup Y is frequently observed in elderly and is associated with flu and respiratory manifestations such as bacteremic pneumonia [4, 12]. Secondary invasive meningococcal infections have been reported to occur 7 to 10 days after flu infections [4, 6]. Interestingly, sepsis was also observed as a common complication during COVID-19 and occurred at a median of 9.0 days (7.0–13.0) after illness onset although the bacterial aetiology was not explored [8]. We therefore explored whether IMD (detectable meningococci in a normally sterile site) was more frequently detected in association with respiratory presentations since the emergence of SARS CoV-2. We screened the NRCMHi database for IMD cases that were associated with “pneumonia” or “bronchopneumonia” as clinical manifestations. A total of 38 cases were detected: 18 cases (14%) in 2020 versus, 13 cases (6%) and 7 cases (4%) in 2018 and 2019 (*P *= 0.029 and *P *= 0.002 using Chi Square Test) respectively. The comparison between the year 2020 and 2019 is highly reliable as the seasonal flu peak occurred in both years at week 6 while in the year 2018 the season flu was more extended with a wide peak over the period between week 01 and week 10 [13]. Several of the 2020 IMD cases were preceded by clinically suspected SARS-CoV-2 infection (Additional file 1: Table S1). These cases were not reported elsewhere.

Age and sex distributions did not differ significantly during this period for the 3 years for IMD cases without respiratory manifestations (Table 1). However, the age distribution differed significantly when respiratory presentations were present compared to cases without respiratory manifestations for each year. Indeed, median ages for IMD cases without respiratory manifestations were 20.7, 21.1 and 20.5 for 2018, 2019 and 2020 respectively (Table 1). These median ages for IMD cases with respiratory manifestations were 74 (*P *< 0.001 Chi Square Test), 82.2 (*P *= 0.016 Chi Square Test) and 70.7 (*P *< 0.001 using Chi Square Test) for the 3 years respectively) (Table 1). It is noteworthy that more males were present among cases with respiratory manifestations in 2020 compared to 2018 and 2019 (Table 1). Serogroup distribution also differed for cases with respiratory manifestations compared to cases without respiratory presentations. The proportions of serogroups W and Y isolates were significantly higher among IMD cases with respiratory presentations (Table 1) in 3 years underlining the role of these isolates in respiratory forms of IMD [4, 6]. These isolates (in particular serogroup W isolates) belonged mainly to non hyperinvasive genetic lineages (Fig. 1 and Additional file 1: Table S1).

The lockdown seems to have reduced inter-human meningococcal transmission that was associated with significantly lower number of “usual” IMD cases compared to the corresponding periods of 2018 and 2019. This decrease involved hyperinvasive but not the non-hyperinvasive isolates further underlying that the former may show higher transmission rates. Moreover, clinical forms with respiratory manifestations seem to increase on the basis of the non-hyperinvasive isolates that may be carried for longer period although of lower virulence. Our data suggest that the increase in these respiratory forms of IMD was concomitant with the COVID-19 pandemic and was mainly observed among elderly and more frequently among males similarly to severe COVID-19 cases [14]. More investigations are required to explore whether these data reveal an enhanced susceptibility to IMD that may be directly linked to the SARS-CoV-2 infections [15]. An additional interference between COVID-19 and IMD may originate from the use of anti-complement drugs that are explored to control COVID-19 by lowering complement mediated pro-inflammatory response [16]. These drugs such as anti-complement compounded 5 (C5) are known to increase the risk for IMD [17].

## Limitations

Several of IMD cases with respiratory presentations corresponded to suspected COVID-19. However, no PCR or serological confirmations of COVID-19 were available for all these cases. Moreover, it is important to underline that our data do not support using or not antibiotics in COVID-19 patients as these questions still require more investigations. Finally, our data correspond to a snapshot of the evolution of IMD epidemiology during a short period of time. Long-term surveillance is required. In the meanwhile, our data highlight the need to enhance surveillance of IMD and to consider large anti-meningococcal vaccination and prophylaxis not only in children but also in elderly.

## Supplementary information

**Additional file 1.** Characteristics of isolates and samples reported in this work.

## Data Availability

Data on all cases and all isolates are provided in the Additional file 1: Table S1.

## Abbreviations

COVID-19: Corona virus disease 2019
IMD: Invasive meningococcal disease
MLST: Multilocus sequence typing
NRCMHi: National reference centre for meningococci and *Haemophilus influenzae*
SRAS-CoV-2: Severe acute respiratory syndrome coronavirus 2
WGS: Whole genome sequencing
